# Effects of TRPV1 Activation by Capsaicin and Endogenous N-Arachidonoyl Taurine on Synaptic Transmission in the Prefrontal Cortex

**DOI:** 10.3389/fnins.2020.00091

**Published:** 2020-02-07

**Authors:** Mingyue Zhang, David Ruwe, Roja Saffari, Mykola Kravchenko, Weiqi Zhang

**Affiliations:** Department of Psychiatry and Psychotherapy, University of Münster, Münster, Germany

**Keywords:** GABAergic inhibition, glutamatergic, NAT, PFC, TRPV1, taurine

## Abstract

While the transient receptor potential vanilloid 1 (TRPV1) ion channel, a non-selective calcium-permeable cation channel with high Ca^2+^ permeability, mainly integrates physical and chemical stimuli for nociception, recent studies suggest that it has a role beyond a noxious thermal sensor. In fact, TRPV1 is presently being considered as a target for treating pathophysiological processes including pain, fear, and anxiety disorders. Although this ion channel has many potential roles, its underlying mechanism of action remains elusive. Here we show in mice that activation of TRPV1-, by the exogenous agonist capsaicin-, regulates synaptic activity in both glutamatergic and GABAergic synaptic transmission. Moreover, activation by the endogenous activator N-arachidonoyl taurine (NAT), induced similar effects as capsaicin. On the other hand, taurine, the decomposition product of NAT, strongly depressed the evoked glutamatergic synaptic transmission. In addition to these findings, we also show the immunohistochemical distribution of TRPV1 in the prefrontal cortex (PFC) of mice, as such studies are currently less frequent in the PFC. Overall, these observations allow for a better understanding of how TRPV1 helps regulate excitatory and inhibitory synaptic activity in the PFC of mice.

## Introduction

The prefrontal cortex (PFC) is crucial for central cognitive control. In particular, the PFC likely orchestrates brain interactions between a wide range of brain structures, which could provide a foundation for the complex forms of behavior observed in animals (Miller, 2000). Specific subregions of the medial PFC (mPFC), namely the infralimbic (IL) and the prelimbic (PrL) cortex are known to play unique roles in fear learning and extinction (LeDoux, 2003). The PFC executes this opportunity by receiving enormous connections from other cortical and subcortical brain structures and produces an appropriate response on the basis of memories and regulating the expression of these memories is critical for mental health (Sotres-Bayon et al., 2006).

The transient receptor potential vanilloid subtype 1 channel (TRPV1) possesses a unique role in thermal sensing, in the perception of pain associated with peripheral inflammation (Wu et al., 2010 TRP ion channel), whereby TRPV1 is activated in response to Aβ-induced degradation hippocampal neuron function and gamma oscillations, after which it helps restore hippocampal functioning (Belleza-Tapia et al., 2018). While TRPV1 are in the CNS widely distributed in the dorsal root ganglia of rats and in the dorsal horn of the spinal cord of pigs (Szallasi and Blumberg, 1991; Helliwell et al., 1998), most prominently along the midline of the posterior hypothalamus and rostral midbrain, which was found in TRPV1 reporter transgenic mice (Cavanaugh et al., 2011), however, the extent of TRPV1 expression and distribution in the PFC is still not well understood.

Kasckow et al. (2000) demonstrated reduced anxiety-like behavior in rats after systemic injection of the TRPV1 antagonist capsazepine (CPZ). In line with this, TRPV1-deficient mice display reduced anxiety and conditioned fear compared with their wild type littermates (Marsch et al., 2007). Furthermore, microinjection of CPZ into the ventral portion of the mPFC enhanced anxiolytic effects (Aguirar et al., 2009). The same results were also observed after injection of Δ9-tetrahydrocannabinol (THC) and the anandamide (AEA) analog methanandamide into the mPFC of rats (Rubino et al., 2008). This phenomenon occurs in a biphasic fashion and supports the current opinion that anxiolytic and anxiogenic effects are mediated by cannabinoid receptor type 1 (CB_1_) and TRPV1, respectively (Rubino et al., 2008). Additionally, the signaling pathways of TRPV1 and CB_1_ have been shown to interact in the PFCs of Swiss mice. Endocannabinoids show a strong a structural relationship to N-acyl amino acids including N-arachidonoyl taurine (NAT), but the functions of NAT remain largely unknown, although in general, N-acyl taurines are weak activators of several transient receptor potential channels. Furthermore, degradation of N-acyl taurine leads to the production of fatty acids and taurine (TAU), and taurine is a well-known inhibitory neuromodulator of GABA receptors (Kontro and Oja, 1987), yet the effect of taurine on synaptic transmission remains to be explored.

On the cellular level, the activation of TRPV1 results in an increase in the frequency of spontaneous excitatory postsynaptic currents (sEPSC) in substantia gelatinosa of adult rat spinal cord slices without altering inhibitory synaptic transmission (Yang et al., 1998). Other reports showed comparable observations of TRPV1 action on glutamatergic transmission in the substantia nigra (Marinelli et al., 2002) as well as in the locus coeruleus, where a presynaptic effect of TRPV1 activation was suggested (Marinelli et al., 2003). Further studies showed that TRPV1 mainly exerts its influence on the excitatory transmission and is associated with increased calcium influx (Doyle et al., 2002; Li et al., 2004; Xing and Li, 2007). However, with help of TRPV1 knockout mice, Benninger et al. (2008) suggest that TRPV1 receptors may not be target molecules for capsaicin regulating glutamatergic synapse in the hippocampus, argue against the hypothesis that capsaicin modulates excitatory synaptic transmission by activating TRPV1 receptors.

Considering the current contradicting pharmacological data in the literature, we carried out a series of experiments to investigate the synaptic effects of TRPV1 in the PrL of mice and expression of TRPV1 in PFC. We further tested the effect of NAT as an endogenous activator for TRPV1 as well as the effect of taurine (a NAT degradation product) to explore the mechanisms underlying TRPV1 regulation of glutamatergic transmission in the mPFC of mice. Our results suggest that the NAT-mediated effect via TRPV1 activation played a critical role in the modulation of synaptic activity of the PFC.

## Materials and Methods

The experiments were performed in accordance with the European Communities Council Directive (86/EEC), and were approved by the Federal State Office for Consumer Protection and Food Safety of North Rhine-Westphalia, Germany. Every effort was made to reduce the number of animals used in the experiments. All mice were given *ad libitum* access to water and food and were housed under a 12 h light/dark cycle. For analyzing *ex vivo* isolated but functional neuronal networks, brain preparations including the mPFC from 8- to 12 week old C57BL/6 mice were employed.

### Slice Preparation and Whole-Cell Recordings

The slice preparation was performed as described previously (Teng et al., 2013). Briefly, after quick decapitation, mice brains were transferred to ice-cold oxygenated artificial cerebrospinal fluid (ACSF). Then, 300-μM-thick slices containing PrL and IL were cut on a vibratome (Leica, 1200) and obtained as previously described. Slices were placed in the recording chamber, which was superfused (4 ml/min) with ACSF at room temperature (RT).

Whole-cell recordings were made in acute coronal PFC slices from prelimbic cortex at a holding potential of -70 mV. The bath solution in all experiments consisted of 125 NaCl, 2.5 KCl 1.25 Na_2_HPO_4_, 2 MgSO_4_, 26NaHCO_3_, 1.5 CaCl_2_, 14 glucose (pH 7.4, aerated with 95% O_2_, 5% CO_2_). The pipette solution contained 140 KCl, 1 CaCl, 10 EGTA, 2 MgCl_2_, 0.5 Na_2_-ATP, and 10 HEPES (in mM); pH was adjusted to 7.2 with KOH.

Spontaneous excitatory postsynaptic currents were detected from pyramidal neurons in PrL layer V in the presence of strychnine (a glycine receptor antagonist; 5 μM) and (-)-bicuculline methochloride (a competitive GABA_A_ receptor antagonist; 5 μM unless otherwise indicated). Miniature GABAergic (mIPSCs) currents were recorded in the presence of CNQX (10 μM), DL-AP5 (50 μM) and 1 μM tetrodotoxin (TTX). Electrically evoked glutamatergic postsynaptic currents (eEPSCs) were recorded from PrL pyramidal neurons in layer V and evoked by 0.1 Hz using the bipolar platinum electrode placed on the PrL cortical layer II/III to stimulate the ascending feed-forward projections to pyramidal neurons in layer V. To record eEPSCs, the pipettes (input resistance: 3–5 MΩ) were filled with the following solution (in mM): 140 potassium gluconate, 1 CaCl_2_, 10 EGTA, 2 MgCl_2_, 4Na_3_ATP, 0.5 Na_3_GTP, 10 HEPES, pH 7.3. Peak amplitudes were averaged from 15 consecutive responses. The input resistance was checked by the current responses to a -10 mV voltage step (20 ms) from a holding potential of -70 mV before every fifth stimulus. For pharmacologically isolated AMPA-or NMDA-mediated EPSCs, we blocked NMDAR with 50 μM AP5 and AMPAR with 10 μM CNQX and by a holding potential of +40 and -70 mV, respectively.

In all experiments, the distance and location between the stimulation and recording electrodes was similar between slices of the different mice, and we could not find any significant difference in latency of evoked responses between the mice. Evoked of EPSCs were all recordings taken at exactly the same elapsed time. Control (Con.) in panel means baseline level before addition of compound. We excluded patches with a serial resistance of >20 MΩ, a membrane resistance of <0.8 GΩ, or leak currents of <150 pA. The membrane currents were filtered by a four-pole Bessel filter at a corner frequency of 2 KHz and digitized at a sampling rate of 5 KHz using the DigiData 1322A interface. Data acquisition and analysis were performed using commercially available software (pClamp10.1; Axon Instruments/molecular Device). Mini Analysis 6.0.3 (Synaptosoft, Decatur, GA, United States) was used to analyze amplitude and frequency of sEPSCs, mEPSCs and mIPSCs), and Prism 5 (GraphPad Software Inc., CA, United States). For drug application, NAT was obtained from Cayman Chemical (Cayman, Chemical, United States). All other chemicals were obtained from Sigma-Aldrich (Sigma-Aldrich, Germany).

### Tissue Preparation for Immunohistochemistry

Altogether, six male C57BL/6 mice (10–16 weeks old) were deeply anesthetized with intraperitoneally injection of 75 mg/kg of ketamine 10% (Bela-Pharm GmbH, Germany) and 3.5 mg/kg xylazine 2% (Serumwerk, Germany) and were transcardially perfused with 0.1 M phosphate buffered saline (PBS, pH 7.0) followed by a fixation procedure using 4% paraformaldehyde (PFA). After decapitation, the brains were quickly removed and kept in 4% PFA overnight at RT and then transferred to 30% sucrose dissolved in PBS for at least 3 days. Serial coronal sections of 25-μm thickness were prepared using a cryostat (Leica CM3050 S) for subsequent immunohistochemistry procedures.

### Immunohistochemistry

Free-floating sections were washed in PBS and then treated with 3% H_2_O_2_ for 30 min, rinsed 3–5 times in PBS, and preincubated in PBS containing 5% normal goat serum. After preincubation, sections were incubated with polyclonal primary antibody anti-TRPV1 (ab31895, Abcam, dilution: 1:500) overnight at 4°C. The next day, sections were rinsed five times in PBS and then incubated for 1–2 h at RT with secondary antibodies including biotinylated goat anti rabbit lgG (BA10001, Vector laboratories, dilution: 1:200). After washing, the sections were incubated with a vectastain ABC complex (PK-4000, Vector laboratories) for 30 min, rinsed again 3–5 times in PBS, developed for 4–6 min in diaminobenzidine (DAB Peroxidase substrate kit SK-4100. Vector Laboratories). After the final rinsing in PBS, the developed sections were wet-mounted on glass slides. Following an air-drying process, sections were cover-slipped with Dako Faramount Aqueous Mounting Medium (S3025, Dako).

### Immunofluorescent Staining

For double-immunofluorescence of TRPV1 and PSD95 (a marker for post synaptic mechanisms), free-floating sections containing PFC were washed in PBS and then preincubated in PBS containing 5% normal goat serum for 3–4 h at RT. After preincubation, sections were incubated with polyclonal rabbit anti-TRPV1 antibody (ab31895, Abcam, dilution: 1:500), and mouse monoclonal antibody anti-PSD95 (ab13552; Abcam; 1:500) for 24 h at 4°C, after which they were rinsed 3–5 times in PBS, the sections were then incubated for 1–2 h at RT in goat anti-rabbit IgG Alexa Fluor 488 (ab150077; Invitrogen; 1:1500) and goat anti mouse IgG Alexa Fluor 647 (ab150115; Abcam; 1:1500) dissolved in blocking solution. Following the final rinsing, the slices were wet mounted onto subbed slides and subsequently dried and cover-slipped with Dako Faramount Aqueous Mounting Medium (Dako; S3025). For fluorescence imaging, tissues were visualized under ab epifluorescence IX81 microscope (Olympus), and for confocal imaging a 700-AX10 laser scanning microscope (Carl Zeiss) was used.

### Data Analysis

The detection threshold of spontaneous and miniature excitatory events was set at twice the baseline noise. To exclude false events, each measurement was visually inspected and analyzed. Subsequently, the measurements were grouped and reported as mean ± standard error of mean (SEM). The number of slices employed in the individual experiment was indicated in brackets. Student’s *t*-test or non-parametric Mann–Whitney test were used to determine differences between data samples for normally- and non-normally distributed data, respectively. Data on drug effects of miniature PSCs were analyzed by paired test. Data on the time course of the capsaicin and NAT effect on sEPSCs were analyzed by one-way ANOVA. Statistical significance is indicated as ^∗^ for *p* < 0.05, ^∗∗^ for *p* < 0.01, ^∗∗∗^ for *p* < 0.001.

## Results

### Capsaicin Altered Glutamatergic Synaptic Transmission in the PrL

**FIGURE 1:**
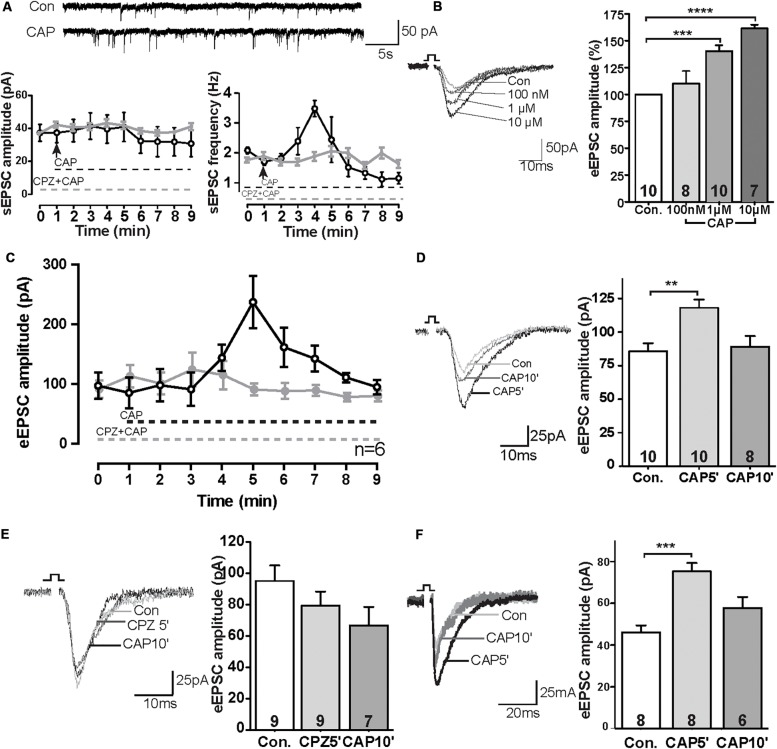
TRPV1 activation altered excitatory synaptic transmission in the PrL. **(A)** Sample trace and analysis of spontaneous sEPSC in the presence of 1 μM CAP. **(B)** Sample trace and dose-dependent effects of CAP on eEPSCs. **(C)** Time course of the effect of CAP (1 μM) on eEPSC in the presence and absence of TRPV1 channel antagonist (CPZ). **(D)** Sample traces and time-dependent effects of CAP (1 μM) on eEPSC. **(E)** Pre-incubation of 10 μM CPZ prevented the CAP effect on eEPSCs. **(F)** In the presence of 50 μM picrotoxin, CAP showed the same effects on eEPSCs as compared to the application of bicuculline methochloride; **p < 0.01, ***p < 0.001, ****p < 0.0001.

We first tested sEPSCs in layer V pyramidal neurons of the PrL. Bath application of capsaicin (CAP, 1 μM) caused a rapid increase of sEPSC frequency of about ∼175% compared to baseline levels (Figure 1A; control: 1.99 ± 0.14 Hz, 3 min after CAP: 3.48 ± 0.26 Hz), whereas the amplitude of sEPSCs was not significantly changed. The rapid CAP-effect remained for a few minutes and decreased rapidly following sustained exposure. Potentiation by CAP is prevented in the presence of the TRPV1 channel antagonist capsazepine (CPZ: 10 μM) (*n* = 6, P > 0.05) (Figure 1A).

To further evaluate the CAP-effect on EPSCs, evoked EPSCs were recorded in pyramidal neurons in the PrL layer V upon II/III stimulation in the presence of different concentrations of CAP. As shown in Figure 1B, application of CAP caused an increase in amplitude of eEPSC in a dose-dependent manner (Figure 1B; Con., 100 nM 110%, ns; 1 μM 143%, *p* < 0.01; 10 μM: 161%, *p* < 0.01). Similar to Figure 1A, application of CAP caused an increase in amplitude of eEPSCs; Preincubation of the slices with the CPZ largely prevented the effects of CAP on the amplitude of eEPSCs during the 10 min recording (Figure 1C). The amplitude of eEPSCs at *t* = 5 min after application (CAP5′) was significant increased, but the effect was absent at *t* = 10 min (CAP10′) (Figure 1D; control: 85.7 ± 5.9 pA, 5 min after CAP: 118.1 ± 6.0 pA, *p* < 0.01; 10 min after CAP, 89.1 ± 8.0 pA, ns; Figure 1E; control: 95.0 ± 9.4 pA; CPZ5′: 79.3 ± 8.5 pA, ns; CAP10′ (1 μM): 66.7 ± 10.9 pA, ns, CPZ: 10 μM; an antagonist of the CAP receptor).

To exclude a possible off-target effect of bicuculline, PFC slices were treated with another GABA_A_ receptor antagonist picrotoxin (50 μM). In this case, the CAP- effect on eEPSC amplitudes remained (Figure 1F), similar to that in the presence of bicuculline (Figure 1C).

### Expression of TRPV1 Channels in the PrL and the Effect of TRPV1 Activation on Central Synapses

**FIGURE 2:**
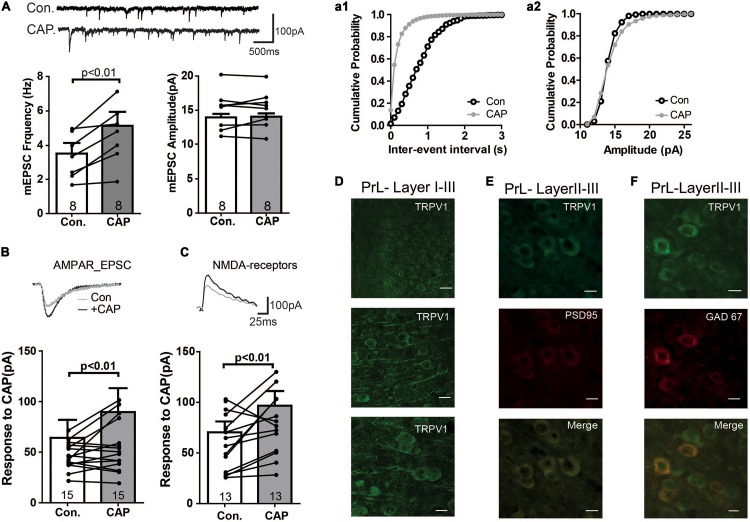
Immunofluorescence study of TRPV1 and its effects on mEPSC in the PFC **(A)** Sample traces, frequencies and amplitudes of mEPSCs before and after CAP treatment. **(a1, a2)** cumulative plots of mEPSCs frequencies and amplitudes **(B,C)** AMPAR- or NMDAR-mediated currents were altered in CAP treated PFC slice. **(D)** Staining of TRPV1 in layers I–III of the PrL. Scale bars: 10 μm. **(E)** Double- staining of TRPV1 and PSD95 in the PrL. **(F)** Double- staining of TRPV1 and GAD67 in the PrL.

The above data suggest the presence of TRPV1 channels and that this channel mediates current in PFC. Therefore, we next directly analyzed the expression of TRPV1 channels in the PrL. As shown in Figure 2A (see also Supplementary Figure S1A), TRPV1 was detectable mainly in the soma of pyramidal-like neurons in the PrL, suggesting that TPRV1 affects glutamatergic neurons. Next we measured glutamatergic neurotransmission: As shown in Figure 2A, the application of CAP enhanced the frequency, but not the amplitude of miniature EPSCs (Con: 3.5 ± 1.6 Hz, CAP: 5.1 ± 2.2 Hz, *p* < 0.01). To further dissect the effect of CAP on glutamatergic synaptic transmission, we next separately measured AMPA- and NMDA-mediated EPSCs in pyramidal neurons of the PrL. Here, NMDA- and AMPA-mediated EPSCs were recorded in layer V pyramidal neurons of the PrL using a holding potential of +40 and -70 mV, respectively. As shown in Figures 2B,C, both AMPA and NMDA EPSCs were significantly increased after CAP treatment (AMPA: 75.9 ± 20.3 pA, AMPA + CAP: 97.4 ± 27.8 pA, *p* < 0.01; NMDA: 75.1 ± 11.8 pA, NMDA + CAP: 100.4 ± 16.6 pA, *p* < 0.01).

We further investigated the subcellular localization of TRPV1 by double labeling neurons with antibodies against TRPV1 and PSD95. As shown in Figure 2E, TRPV1 may be enriched in the postsynaptic density and was associated with PSD95 in PrL neurons (see also Supplementary Figures S1B,C), supporting the idea that TRPV1 directly affects the glutamatergic terminal in the PrL.

**FIGURE 3:**
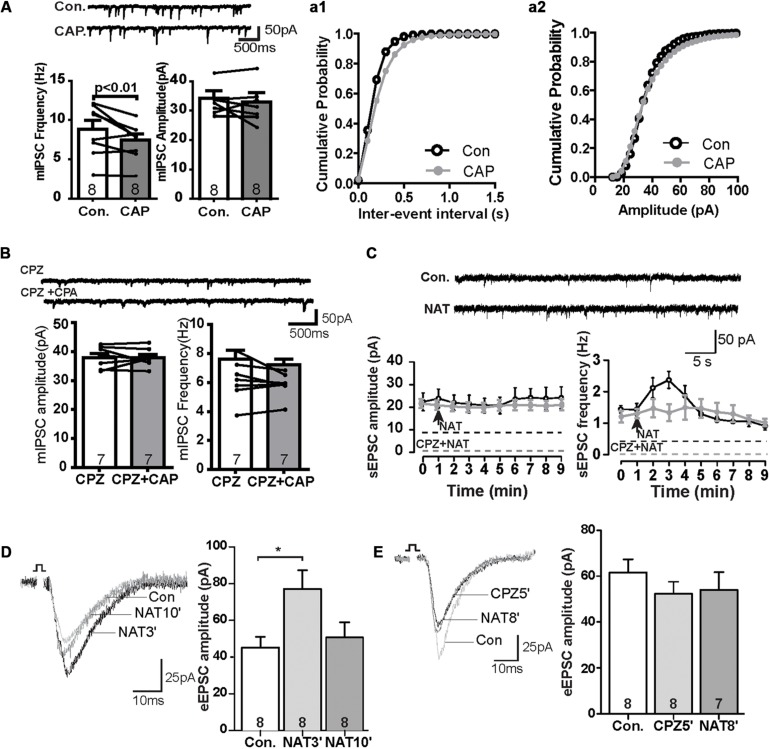
The effect of CAP reduced frequency of mIPSC and NAT activation displayed similar synaptic responses as CAP. **(A)** Sample traces, frequencies and amplitudes of mIPSCs before and after CAP treatment. **(a1, a2)** Averaged cumulative frequency and amplitude distribution plots of mIPSC amplitude **(B)** Representative traces for mIPSC frequency and amplitude under CPZ and CAP conditions. **(C)** Sample traces, frequency and amplitudes of sEPSCs before and after NAT treatment and time course of the effect of NAT on sEPSC in the presence and absence of CPZ. **(D)** Sample traces and quantitative analysis of eEPSC data before and after application of NAT. **(E)** Sample traces and quantitative analysis of NAT-effect on eEPSCs in the presence of CPZ; *p < 0.05.

We next also wanted to know whether TRPV channels express in the GABAergic interneuron. We performed the immunofluorescence staining with the help of GAD67-GFP^+^ mice, where GFP was expressed under the promoter of the glutamic acid decarboxylase gene. As shown in Figure 2F, TRPV1 was expressed in all GFP-positive neurons (see also Supplementary Figures S1D–F), indicating that TRPV1 might also affect the GABAergic synaptic transmission. To further test this idea, we measured mIPSCs. Interestingly, application of CAP decreased the frequency of mIPSCs, but not their amplitude in the PrL (Figure 3A). The TRPV1 channel antagonist prevented the decrease in mIPSCs, verifying the involvement of TRPV1 channel. Altogether, these results demonstrate that TRPV1 activation modulate both glutamatergic and GABAergic neurotransmission in the PrL (Figure 3B).

### N-Arachidonoyl Taurine (NAT) Enhances the Glutamatergic Synaptic Transmission, Similar to Capsaicin

In the kidney, NAT displays agonist properties on TRPV, and its abundance is regulated analogously to anandamide by FAAH (Saghatelian et al., 2006). In the PrL, NAT (10 μM) did not affect the amplitude of sEPSCs, but rapidly increased the frequency to about 130% of baseline levels within the first minute after its application. This effect reached a maximum of approximately 150% at *t* = 2 min (control: 1.4 ± 0.2 Hz, NAT at *t* = 2 min: 2.4 ± 0.3 Hz, Figure 3C) and gradually subsided afterward. The increase of the sEPSC frequency so quickly could suggest that a TRPV1 might have a higher affinity for NAT than CAP (c.f. Figure 1A). The effects of NAT on TRPV1 channel was blocked in the presence of antagonist CPZ (Figure 3C). Furthermore, the amplitude of eEPSCs was significantly increased at *t* = 3 min (NAT3′) (Figure 3D; control: 45.2 ± 5.4 pA, NAT3′: 77.1 ± 9.6 pA; *p* < 0.05) but not at *t* = 10 min (Figure 3D; NAT10′: 50.7 ± 7.7 pA). Similar to Figure 1E, pre-incubation of slice with CAZ during the whole recording prevented the NAT -effect on eEPSCs at *t* = 5 min (CPZ5′) and *t* = 8 min (NAT8′) (Figure 3E; control: 61.6 ± 5.3 pA, NAT8′: 54.1 ± 7.1 pA, n.s.), suggesting that NAT enhanced eEPSCs by activating the TRPV1 receptor channels.

### Taurine Depressed Glutamatergic Synaptic Activity Independent of TRPV1 Channels

**FIGURE 4:**
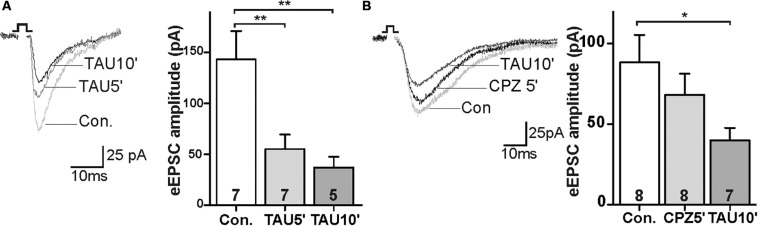
Taurine depressed glutamatergic synaptic transmission independent of TRPV1 activation. **(A)** Sample traces and quantitative analysis of eEPSCs data before and after application of TAU. **(B)** Sample traces and quantitative analysis of the TAU-effect on eEPSCs in the presence of CPZ; *p < 0.05, **p < 0.01.

FAAH regulates NAT degradation, leading to the production of fatty acids and taurine (TAU; Long et al., 2011). We next tested the synaptic response to the taurine. The amplitude of eEPSCs were decreased in the TAU treated slices within 5 min of incubation (Figure 4A; control: 143.1 ± 25.7 pA, TAU5′: 55.0 ± 13.1 pA; *p* < 0.01). This effect persisted during the drug exposure (Figure 4A; control: 143.1 ± 25.7 pA, TAU10′: -45; *p* < 0.01). Furthermore, CPZ was unable to block the TAU effect on eEPSCs in the PrL (Figure 4B; control: 88.2 ± 15.9 pA, CPZ5′: 68.2 ± 12.2 pA, ns; TAU10′: 39.9 ± 7.1 pA; *p* < 0.05). These data suggest that TAU-mediated suppression of glutamatergic synaptic activity is independent of TRPV1 receptors.

## Discussion

The current study provides that capsaicin and NAT, two known activators of TRPV1 channels, have physiological effects on neurons in acute slices from mouse prelimbic cortex. The most thoroughly explored effect was an increase in the EPSC amplitude in layer V pyramidal neurons evoked by stimulating in layer II/III and in the frequencies of sEPSCs and mEPSCs in layer V pyramidal neurons. The effects peaked within a few minutes and then desensitized. They were generally similar between the two compounds to the extent that they were compared directly. The effects were observed at concentrations that are consistent with known TRPV1 potencies of these compounds, and in some cases the effects were shown to be blocked by pre-treatment with capsazepine, which is a selective vanilloid receptor antagonist. Additional evidence of TRPV1 channel expression in prelimbic cortex provided that TRPV1 may be present in inhibitory neurons as well as excitatory neurons. Finally, it was found that taurine, a metabolic derivative of NAT, tended to reduce the evoked EPSC amplitude, but this effect was not blocked by capsazepine.

Aguirar et al. (2009) demonstrated that microinjection of CPZ into the ventral portion of the mPFC resulted in an anxiolytic-like effect, suggesting that TRPV1 activation in the PrL would enhance anxiety-like behavior. Indeed, our results demonstrated that the activation of TRPV1 channels both by CAP and NAT enhanced the excitatory synaptic transmission in the PrL (Figure 1A), in line with the top-down regulatory function of PrL associated with the amygdala and nucleus accumbens in the expression of aversive and appetitive behavior (Peters et al., 2009). This notion is supported by studies of TRPV1 KO mice in which show less anxiety-related behaviors, less freezing, less contextual fear and reduced LTP (Marsch et al., 2007). Consistently, we demonstrated that activation of TRPV1 has effect on AMPAR-or NMDAR-mediated currents in excitatory neuron in adult PFC slices (Figures 2C,D). Meanwhile, we observed CAP increased the frequency of sEPSCs and mEPSCs, in agreement with report that CAP enhanced the frequency of spontaneous and miniature excitatory synaptic currents, suggesting that the effect of CAP on excitatory synaptic current is possible via an action potential-independent release probability (Thakre and Bellingham, 2017).

In contrast to its action on glutamate transmission, activation of TRPV1 channels by capsaicin had also effect on the frequency of mIPSCs. Furthermore, immunohistochemistry data suggested that TRPV1 may be present in inhibitory interneuron. Consistently, Hurtado-Zavala et al. (2017) showed that TRPV1 is expressed in oriens-lacunosum-moleculare (OLM) interneurons in the hippocampus, and promote excitatory innervation, however, we observed reduced frequency of mIPSCs. It is likely that by activation of TRPV1 leading to produce magnitudes of calcium influx and further production of endocannabinoid (eCB). Typically, eCB are mobilized from the postsynaptic compartment and act retrogradely to suppress synaptic transmission (Chávez et al., 2014). Another study indicates that CAP selective and significantly decreases sIPSCs amplitude and not frequency (Thakre and Bellingham, 2017). However, we observe the decrease of the frequency and not amplitude of mIPSCs. We speculate that different CAP effects may be attributed to variation in brain regions [hypoglossal motor neurons (HMN) in Brainstem in rats vs. Pyramidal neurons in (PFC)] or age of rodents (neonatal rat vs. adult mice).

Despite the predominant expectation that capsaicin, the pungent chemical from chili peppers, possesses its agonistic properties on TRPV1 exclusively in the PNS, and despite that only a few endogenous activators of this channel have been profoundly explored with respect to aversive or appetitive behavior, other reasons led us to study NAT. As mentioned earlier, FAAH is the critical enzyme that determines the physiological levels of both anandamide and NAT (Kathuria et al., 2003; Saghatelian et al., 2006). NAT, unlike capsaicin, exists in the brain under physiological conditions. In the current study, we provide electrophysiological evidence that NAT modulates excitatory synaptic transmission in the PFC (Figure 3B), similar to CAP-mediated activation of TRPV1 in the PrL (Figure 3A). Both CAP and NAT elevated the frequency of sEPSCs to a similar extent, but both did not alter the amplitude. Furthermore, the NAT-induced facilitation of sEPSCs appeared faster in comparison to the capsaicin application (Figure 2A vs. Figure 1A). Both effects remained for maximum of 2–3 min and underwent a rapid decrease during sustained exposure, possibly due to desensitization of the receptor in order to prevent sustained excitability (Musella et al., 2009).

As mentioned before, NAT is decomposed by FAAH into taurine, whose role in the pathophysiology of anxiety is quite controversial. Early studies have reported that taurine has anxiolytic-like effects (Zhang and Kim, 2007) or no effects at all (Whirley and Einat, 2008). While one study showed that the effects of taurine on GABA_A_ receptors are linked to the benzodiazepine binding site (Jia et al., 2008), there is evidence for an immediate interaction between the GABAergic system and the vanilloid system in the brain (Manna and Umathe, 2011). GABA receptor associated proteins (GABARAP) exhibit an important contribution to TRPV1 signaling by modulating the channel expression in the plasma membrane and its functional activity at the level of channel gating and desensitization (Lainez et al., 2010). However, this should not be in our case, as the GABAergic transmission was blocked by bicuculline and strychnine in the bath to isolate glutamatergic synaptic currents. In the present study, taurine depressed glutamatergic transmission in the PrL, independent of the activation of TRPV1 receptors (Figure 4A). Recent studies by Moriuchi et al. (2018) show that taurine can inhibit down line linestream signaling induced by capsaicin. Consistently in our studies, taurine acts in a TRPV1 dependent pathway. Moreover, The decrease of amplitude caused by taurine could be complex and may likely involve other modulating systems, including endocannabinoids (Iannotti et al., 2016).

We propose that TRPV1, NAT and taurine are important for modulating synaptic activity in the PFC, and impairment in balance as well as disturbance in synaptic plasticity could be a pathogenic mechanism of psychiatric disorders. According to recent publications, important correlations exist between genetic defects of TRPV1 and anxiety/depression, and among psychotropic drugs, capsazepine seems to have protective effects on psychiatric disorders via TRP channels (Naziroglu and Demirdas, 2015). It is conceivable that our findings could be useful in understanding disorders that involve TRPV1, and this channel may represent a compelling target for therapeutic agents against related psychiatric disorders.

## Data Availability Statement

All datasets generated for this study are included in the article/Supplementary Material.

## Ethics Statement

The animal study was reviewed and approved by the Landesamt für Natur, Umwelt und Verbraucherschutz of the state of North Rhine Westphalia, Germany. Written informed consent was obtained from the owners for the participation of their animals in this study.

## Author Contributions

MZ and WZ initiated and directed the study and wrote the manuscript. DR, RS, and MK performed the research and analyzed the data. All authors contributed to approved the manuscript.

## Conflict of Interest

The authors declare that the research was conducted in the absence of any commercial or financial relationships that could be construed as a potential conflict of interest.

## Supplementary Material

FIGURE S1 Immunoreactivity of TRPV1 Channel in sections of the PFC. Overview of TRPV1-stained coronal section of PFC (**A**, left) and in higher magnification 20× Scale bar: 200 μm and 60× Scale bar: 20 μm. **(A)** TRPV1–IR co-localizes with PSD95 in different layers of the IL **(B,C)**, Scale bar: 10 μm. TRPV1-IR co-localizes with GAD67 in layers of the PrL and IL **(D–F)**,Scale bar: 10 μm. ACC, anterior cingulate cortex; IL, infralimbic cortex; M2, motor cortex 2; Prl, prelimbic cortex.
