# Supplementary figures and images for: Effects of TRPV1 Activation by Capsaicin and Endogenous N-Arachidonoyl Taurine on Synaptic Transmission in the Prefrontal Cortex
Source: Front Neurosci. 2020 Feb 7;14:91. doi: 10.3389/fnins.2020.00091 (PMC7020858; doi:10.3389/fnins.2020.00091)

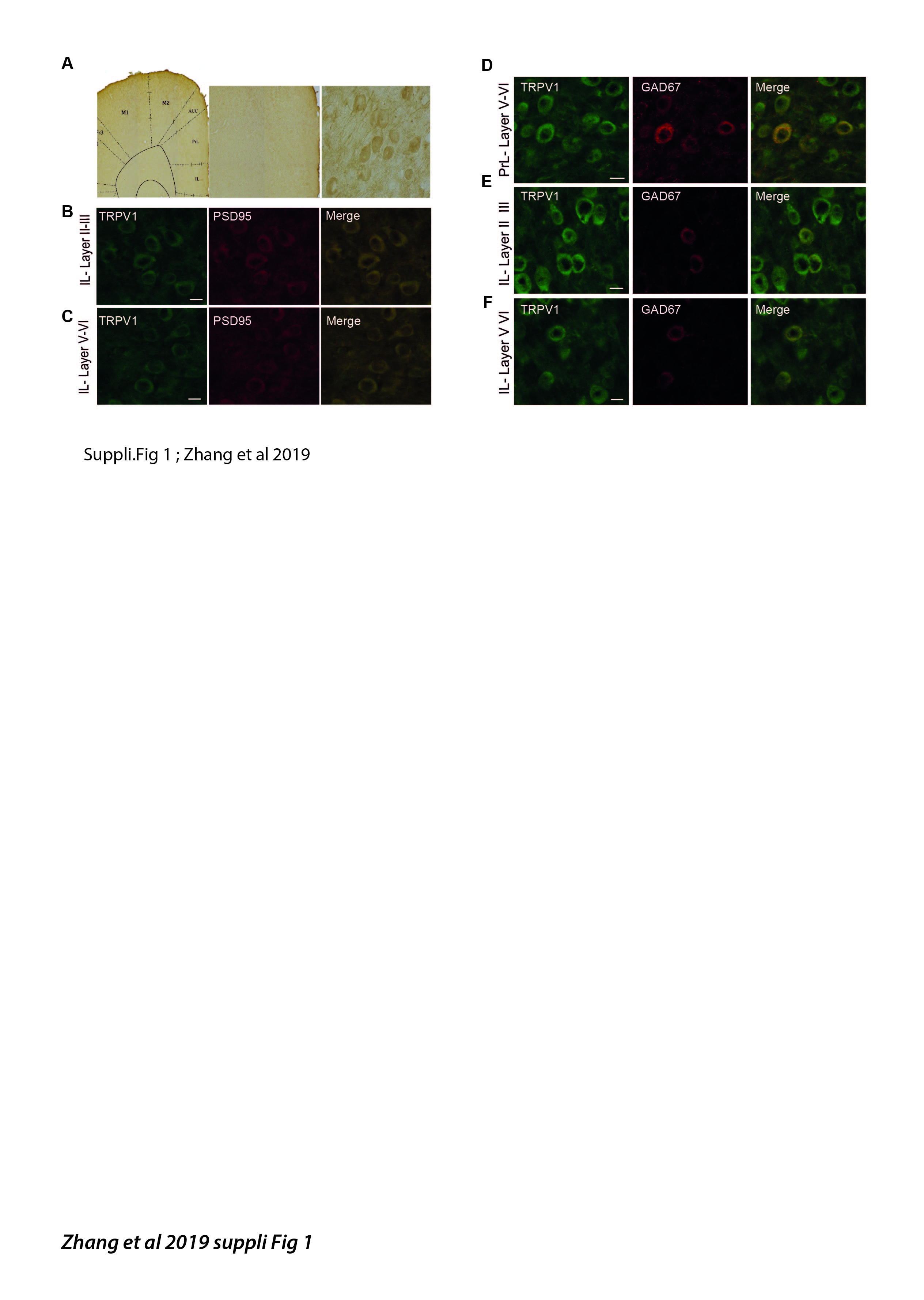

Supplement: FIGURE S1 — Immunoreactivity of TRPV1 Channel in sections of the PFC. Overview of TRPV1-stained coronal section of PFC (A, left) and in higher magnification 20× Scale bar: 200 μm and 60× Scale bar: 20 μm. (A) TRPV1–IR co-localizes with PSD95 in different layers of the IL (B,C), Scale bar: 10 μm. TRPV1-IR co-localizes with GAD67 in layers of the PrL and IL (D–F),Scale bar: 10 μm. ACC, anterior cingulate cortex; IL, infralimbic cortex; M2, motor cortex 2; Prl, prelimbic cortex. [file Image_1.jpg]
